# Endodontic treatment of a two-rooted mandibular first premolar with four root canals: a case report

**DOI:** 10.3389/fdmed.2024.1498167

**Published:** 2024-12-11

**Authors:** Peiling Hu, Shuang Feng, Xin Li, Guangwen Li, Shiting Li

**Affiliations:** ^1^ Department of Endodontics and Operative Dentistry, The Affiliated Stomatological Hospital, Southwest Medical University, Lu Zhou, China; ^2^ Department of Implantology, The Affiliated Stomatological Hospital, Southwest Medical University, Lu Zhou, China

**Keywords:** bicuspid, anatomical variations, cone-beam computed tomography, root canal therapy, dental operating microscope

## Abstract

**Background:**

Mandibular first premolar has a complex and variable anatomy of the root canal system, which often leads to failure of endodontic treatment due to missing root canals. Identifying the complete structure of the root canal system to ensure that all root canals are perfectly cleared and filled becomes critical to the success of root canal therapy. This report introduced a unique case of endodontic treatment of a two-rooted mandibular first premolar in the buccolingual direction with a total of four canals.

**Case presentation:**

An adult male patient with a lower left first premolar was diagnosed with acute apical periodontitis and treated with open pulp drainage in a general hospital. One day later, due to the complexity of the root canal structure, the patient was referred to our clinic for subsequent treatment. The tooth #34 was diagnosed with abnormal central cusp, apical periodontitis, and incomplete fracture through clinical and x-ray examinations. Cone-beam Computed Tomography (CBCT) results showed that the tooth #34 processed two roots with a buccolingual bifurcation and a total of 4 root canals: 1 lingual canal, 2 mesiobuccal canals, and 1 distobuccal canal. Notably, the buccal root presented a C-shaped configuration, and the mesiobuccal canals were of 2-1 type. The tooth was treated with microendodontics and crown restoration. One year after the treatment, the follow-up results showed that the tooth #34 was functioning normally without any abnormalities.

**Conclusion:**

This report enhances our understanding of the anatomical variations in the root canal system of the mandibular first premolar and emphasizes the importance of CBCT in identifying anatomical variations within the root canal system. Clinicians must be aware of such changes in the mandibular first premolar during treatment to ensure a perfect treatment and better prognosis in clinical practice.

## Background

The success of root canal therapy (RCT) is closely related to the degree of root canal morphological variability and the clinician's knowledge of root canal morphology (1). The mandibular first premolar has been reported to be predominantly single-rooted, with occasional reports of two-rooted variants (2, 3), while three-rooted and more than three-rooted variants are extremely rare (4). The root canal system of the mandibular first premolar is also highly variable (5). Aamina et al. reported a case of a mandibular first premolar with three canals (6–10), while Yi Du et al. documented a mandibular first premolar with four canals (11, 12), and Ahmad et al. described a mandibular first premolar with five canals (2, 13). Therefore, due to the complexity and diversity in root canal system, there are many uncertainties in the RCT and long-term prognosis of the mandibular first molars (14). However, the advancements and widespread adoption of diagnostic and treatment technologies and equipment, such as Cone-beam computed tomography (CBCT) and dental operating microscope (DOM), can aid in understanding the frequency of certain variants within the population and significantly improve the outcomes of RCT for teeth with complex root canal anatomies (15). With the patient's informed consent, the objective of this report is to present a rare case of a mandibular first premolar with a double root and a total of four canals, which was successfully managed through the use of CBCT scanning as an adjunctive imaging modality and non-surgical endodontic treatment utilizing a dental operating microscope.

## Case presentation

**Figure 1:**
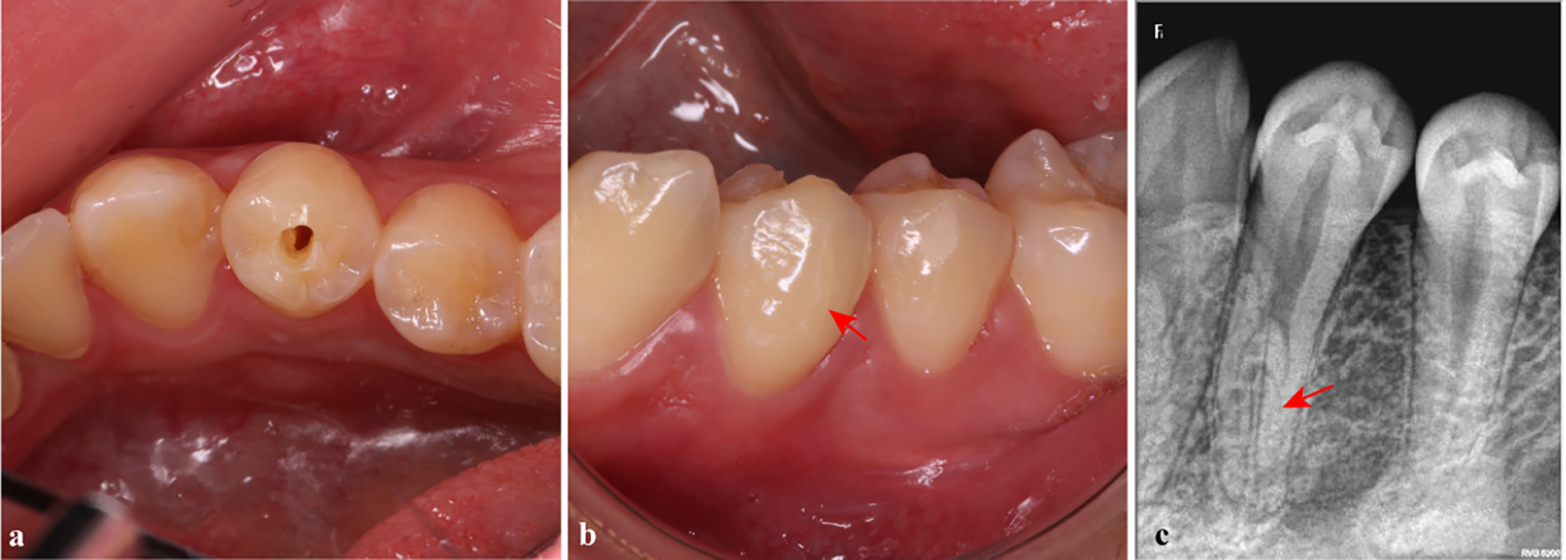
Preoperative examination of tooth #34. **(a)** A probing perforation of the pulp of tooth #34; **(b)** A buccal crack on tooth #34 (red arrow); **(c)** There was an anatomical variation in the lower segment of the root canal on the x-ray image (red arrow).

**Figure 2:**
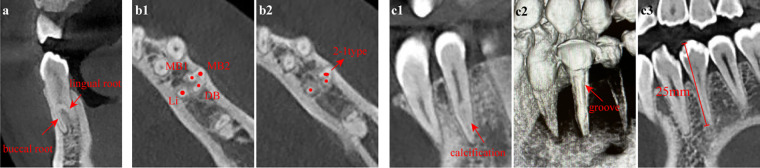
Preoperative CBCT images of tooth #34. **(a)** The root of tooth #34 was buccolingually bifurcated in the middle 1/3 on the coronal plane; **(b1)** There were three canals within the buccal root, while there was only one canal within the lingual root; **(b2)** The two mesiobuccal canals fused into one at the apical region (2-1 type); **(c1)** The apical region of the mesiobuccal root canal was suspected to be calcified and non-patent on the sagittal plane; **(c2)** The buccal root exhibited a C-shaped configuration with a groove; **(c3)** The lingual root contained a single canal with a length of approximately 25 mm. **(a)** coronal plane; **(b)** axial plane; **(c)** sagittal plane. MB1, mesiobuccal1 canal; MB2, mesiobuccal2 canal; DB, distobuccal canal; Li, lingual canal.

The patient is a 26-years-old male without systemic diseases. He had been experiencing intermittent pain during eating in the lower left posterior teeth for half a year, and had been experiencing severe spontaneous pain for the past 3 days. He was diagnosed with acute periapical periodontitis by another hospital and had undergone pulp exposure and drainage treatment. Due to the complex structure of the root canal system, he was referred to our hospital. Clinical examination in our department revealed a malformed central cusp on the occlusal surface of tooth #34, as well as a probing perforation of the pulp (Figure 1a), cracks in the enamel of the buccal surface (Figure 1b), slight tenderness to percussion, no loosening, and no obvious periodontal abnormalities. Diagnostic x-ray showed that tooth #34 had a bifurcation in the middle portion of the root, unclear image of the lower portion of root canal, and slight widening of the periodontal ligament (Figure 1c). CBCT examination showed that the middle third of the #34 tooth root bifurcated into buccal and lingual roots (Figure 2a). The buccal root of the tooth contained 3 canals (Figure 2b1), designated as mesiobuccal 1 (MB1), mesiobuccal 2 (MB2), and distobuccal (DB). The mesiobuccal double canals were configured in a 2-1 pattern (Figure 2b2), with the apical region of the mesiobuccal canals displaying unclear radiographic images (Figure 2c1). The buccal root exhibiting a C-shaped configuration and visible grooves on the root surface (Figure 2c2). The lingual canal (Li), on the other hand, is a single-canal system. The periodontal ligament space in the apical region of the buccal root was widened. The average length of the tooth was approximately 23 mm, with the lingual root being the longest, measuring approximately 25 mm (Figure 2c3). Tooth #34 was diagnosed with a malformed central cusp (a.k.a. dens evaginatus) (16), apical periodontitis, and a cracked tooth (cracked tooth syndrome). The treatment plan included microendodontics and a full crown restoration for tooth #34.

**Figure 3:**
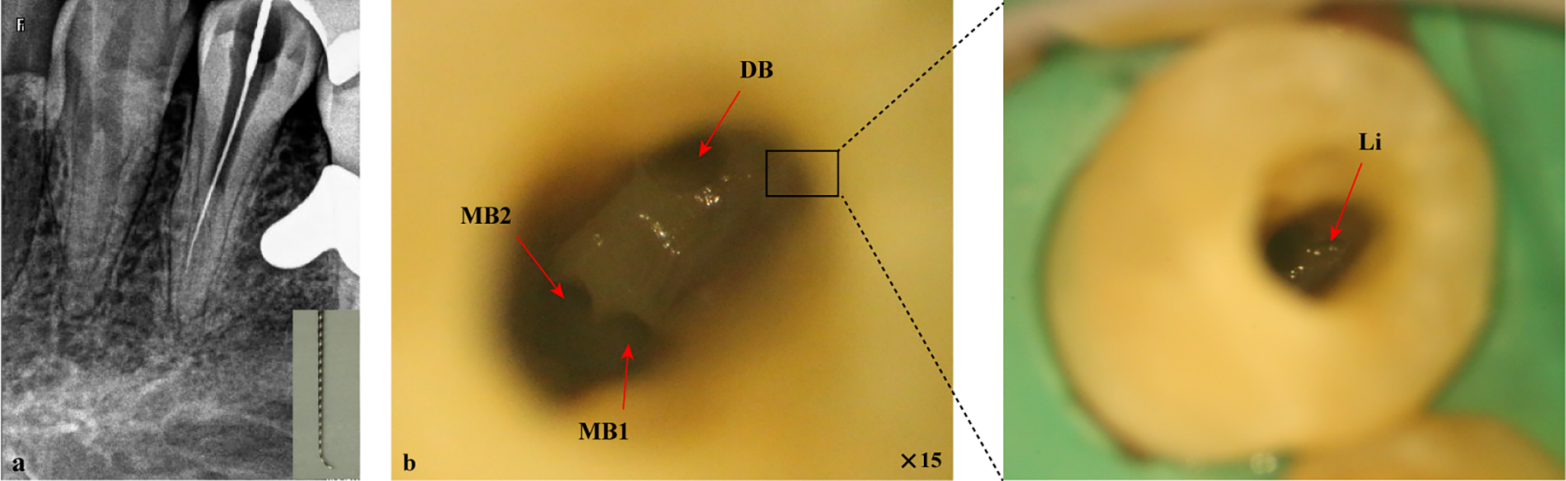
Root canal preparation for tooth #34 under a dental operating microscope. **(a)** X-ray tracing showed that the MB2 canal had reached the working length; **(b)** The four canal orifices were exposed. MB1, mesiobuccal1 canal; MB2, mesiobuccal2 canal; DB, distobuccal canal; Li, lingual canal.

The initial treatment process: The tooth #34 was isolated with a rubber dam, and the pulp cavity was prepared under a dental operating microscope. The ultrasonic ET18 was used to trim the pulp chamber wall and expose the root bifurcation. A total of four root canal orifices were located. Among them, there was a step in the apical 1/3 of MB2, which was wrapped around using a tip pre-curved #8 C+-file (Dentsply, Maillefer, Switzerland) with the aid of ethylenediaminetetraacetic acid (EDTA) gel, and x-ray tracing confirmed that the MB2 canal had reached the working length as shown in Figure 3a. The #8 and #10 K-files (Dentsply) were used to unclog root canals and measure working length (WL) with an electronic apex locator (Dentsply): 23 mm for MB1, 22.5 mm for MB2, 23 mm for DB, and 25 mm for Li. The MB1 and DB canals were prepared to #25 using Waveone Gold (Dentsply). The MB2 and the Li canals were prepared to #25/0.2 taper using K-files (Figure 3b). Every root canal was flushed with 1% sodium hypochlorite + 17% EDTA, ultrasonic irrigation. After drying the root canal, calcium hydroxide paste was filled and temporarily sealed with zinc oxide.

**Figure 4:**
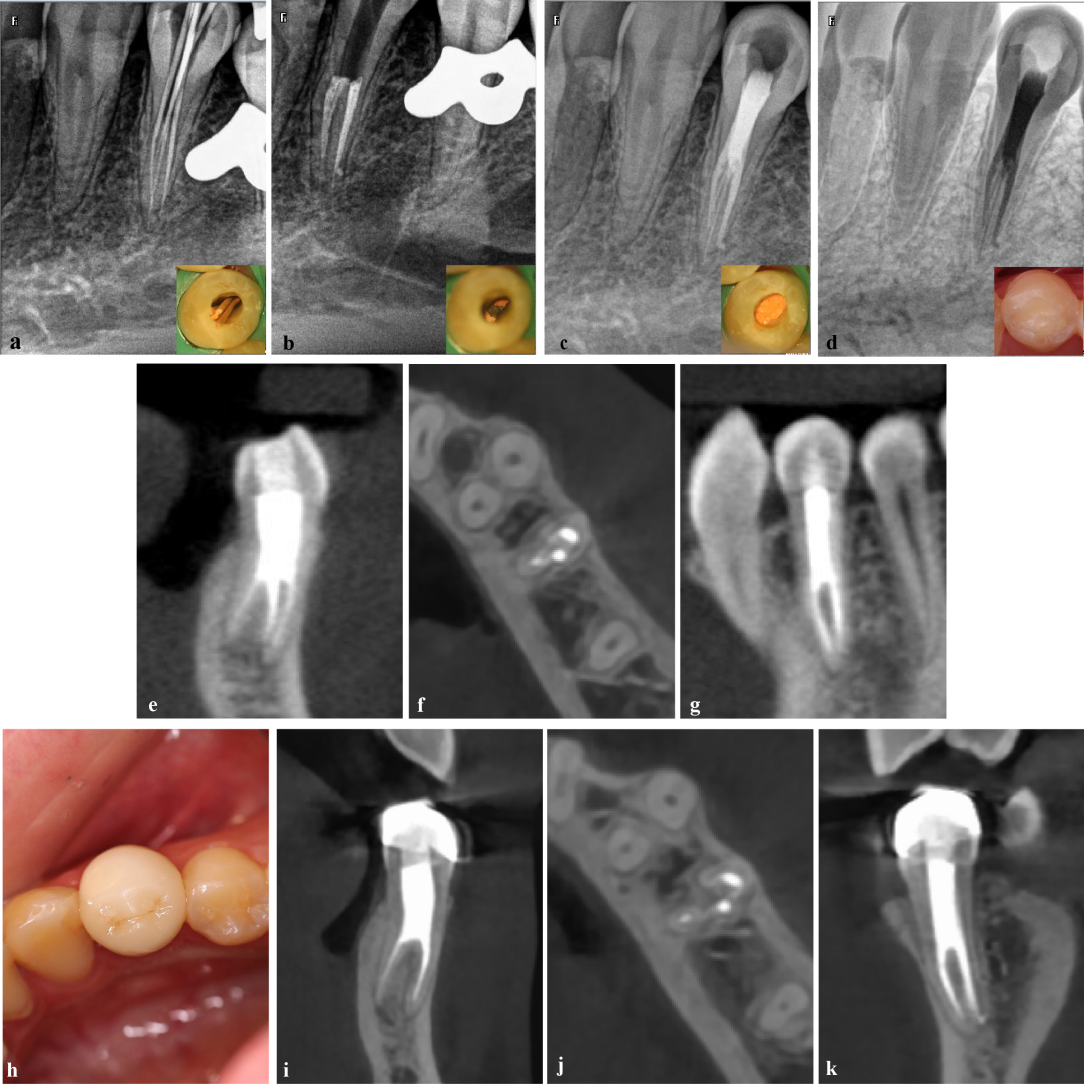
The root canal filling of tooth #34 under a dental operating microscope. **(a)** The canals were trial-fitted; **(b)** The filling of the root canals in the lower segment of the root bifurcation; **(c)** The upper segment was backfilled with gutta-percha; **(d)** The final coronal filling as presented in the inverted x-ray image; **(e–g)** The postoperative CBCT results showed that the root canals were filled densely and continuously without gaps on the coronal plane **(e)**, axial plane **(f)**, and sagittal plane **(g)** Follow-up visit 1 year after RCT. **(h)** The full crown restoration of tooth #34; **(i–k)** CBCT results showed that the four root canals were densely and evenly filled, with no apparent abnormalities observed in the periapical region on the coronal plane **(i)**, axial plane **(j)**, and sagittal plane **(k)**.

The patient reported that the pain had subsided after the 2-week follow-up for teeth. The clinical examination results revealed that the temporary filling on tooth #34 was intact and there was no percussion pain. The treatment process during the follow-up visit: tooth #34 was isolated with a rubber dam, and the root canals were ultrasonically irrigated under DOM. The canals were trial-fitted (Figure 4a). Then the lower segment of the root bifurcation was filled using the single-cone technique with iRoot SP (Innovative BioCreamix, Vancouver, Canada) (Figure 4b), and the upper segment was backfilled with gutta-percha (Figure 4c). A 3M™ Filtek™ Z350 XT Flowable restorative material (3M, St. Paul, USA) was used as a base, and 3M™ Filtek™ Z350 XT resin (3M) was used for the final restoration (Figure 4d) (17). After the root canal filling was completed, the CBCT examination was performed. The CBCT results showed that the filling materials of the four root canals were uniform and dense, and there was no gap between the filling materials and between the filling materials and the root canal walls (Figures 4e–g). The patient had no abnormal condition in the follow-up 2 weeks after the treatment and was sent to the prosthodontics department for full crown restoration. One year after the treatment, the patient returned for a follow-up and reported no discomfort. Clinical examination revealed that the tooth #34 had undergone full crown restoration with good marginal sealing, no percussion pain, no looseness, and no significant periodontal abnormalities (Figure 4h). CBCT results showed that the margin of the 34 tooth's full crown was well-sealed, the 4 root canals were uniformly and densely filled, and there were no significant abnormalities in the periapical region (Figures 4i–k).

## Discussion and conclusions

The prognosis of RCT is influenced by many factors (18), mainly depending on whether the microorganisms in the infected root canal can be successfully removed and prevented from entering the root canal system (19). The complex anatomy of the root canal complicates the process of root canal preparation and makes microbial eradication challenging (20). However, incomplete root canal preparation or missed root canals often lead to the retention of microorganisms (21, 22), which in turn can cause or exacerbate the infection of apical region (23). Karabucak et al. have found that teeth with missing root canals are 4.38 times more likely to develop pathological changes compared to normal teeth. Notably, among molars and premolars that develop apical periodontitis following root canal therapy, the incidence of missing root canals is 83% (24, 25). Within this context, premolars exhibit a relatively high proportion of missing root canals among teeth with apical periodontitis post-treatment, with the highest reported proportion reaching 43% (23). Additionally, Rouhani et al.'s report underscores that in cases of failed root canal therapy, the prevalence of missing root canals is particularly high in the maxillary and mandibular first premolars, with a rate of 100% for the maxillary first premolars and 83.3% for the mandibular first premolars (26). Consequently, the accurate identification and complete clearance of all root canals during root canal therapy are paramount. Achieving this necessitates a comprehensive understanding of the anatomical structure and root canal system of first premolars, which is crucial for ensuring successful treatment outcomes.

Indeed, the complexity of the root and root canal system of the first premolar is significantly underestimated (27, 28). The literatures indicate that there exist varying degrees of variability in the roots and root canal systems of mandibular first premolars across different racial populations (15, 29–33). Studies have shown that in the Chinese population, the majority of mandibular first premolars are single-rooted, accounting for 99.4%, while only 0.6% of these teeth have two roots. Among them, 64.04% have a single canal system, 34.27% have two canals, 1.69% have three canals, and four or more canals are extremely rare (29, 34, 35). The C-shaped canal system, characterized by its complexity and thin isthmus prone to lateral perforation, poses additional challenges to RCT, even when nickel-titanium instruments are used for canal preparation (36). Although C-shaped canal morphology is more common in the Chinese population, typically occurring in the mandibular second molar, its occurrence in the mandibular first premolar is relatively rare, with an incidence rate of 10%–18% (37–39).

Until now, there have been only a few reported cases of successful RCT for mandibular first premolars with anatomical variations (7–10, 13). In these instances, the majority of the mandibular first premolars featured a single root with three, four, or even 5 canals. In 2021, Penukonda et al. documented a case of a mandibular first premolar with two roots that underwent RCT (12). In that particular case, the root of the tooth was divided into two roots in the mesiodistal direction at the middle third, with each root containing two canals. Conversely, in this case report, the mandibular first premolar bifurcated into buccal and lingual roots at the middle third of the root. The lingual root contained a single canal, while the buccal root exhibited a C-shaped configuration with three canals, including a mesiobuccal 2-1 type root canal system, making it significantly more complex than the previous case.

Despite the rarity and challenge posed by the unique number and morphology of the roots, as well as the variant root canal system, we successfully and smoothly completed the RCT of this tooth. The success of the case detailed in this report can be primarily attributed to our prompt recognition of the tooth's anatomical variations in its root canal system, facilitated by preoperative radiographic findings. Our timely and comprehensive assessment of the complex anatomy of tooth #34's root canal system using CBCT enabled us to accurately gauge the treatment's difficulty. Indeed, the utilization of CBCT has proven to be invaluable in obtaining a thorough understanding of the intricate root canal system (40, 41). Preoperative CBCT not only streamlines the RCT process but also markedly reduces the failure rate by allowing for a precise evaluation of the treatment's complexity (42, 43). Studies have shown that CBCT is an effective and precise diagnostic tool for analyzing the mandibular first premolar (44, 45). Furthermore, the incorporation of DOM during the treatment significantly contributed to its successful outcome. The combination of DOM with CBCT has emerged as the preferred approach for both diagnosis and treatment (46). However, in order to preserve more dental tissues, the dentin collar above the lingual canal was not completely removed during the treatment process, which hindered the use of large-taper nickel-titanium instruments for root canal preparation due to inadequate access. This may result in insufficient cleaning and shaping of the root canal, potentially compromising infection control. Therefore, long-term follow-up observation is necessary.

This case report enriches the root and root canal types of mandibular first premolar, which helps clinicians to gain a more comprehensive view of the variations in the root canal morphology of mandibular premolars. This, in turn, enables the adoption of more suitable treatment plans to improve long-term outcome. When treating mandibular first premolars with complex root canal systems, clinicians should first enhance their understanding of root canal variability and routinely utilize CBCT for preoperative assessment to accurately identify the number and morphology of root canals. During treatment, it is recommended to combine the use of DOM to provide a clear visual field, ensuring thorough cleaning and shaping of all root canals, with special attention to anatomical variations such as C-shaped canals. Postoperative follow-up should be closely monitored to promptly detect and address any issues. Additionally, clinicians should continuously learn and update their knowledge to adapt to new techniques and methods in the field of endodontics, enabling them to provide safer and more effective treatment for complex root canal systems.

## Data Availability

The original contributions presented in the study are included in the article/Supplementary Material, further inquiries can be directed to the corresponding author.
